# Strengthening parental capabilities: Consensus-based guidelines for supporting parents of children with developmental disabilities – A modified hybrid Delphi study

**DOI:** 10.4102/safp.v68i1.6321

**Published:** 2026-06-10

**Authors:** Lumka Magidigidi-Mathiso, Jose Frantz, Gerard Filies

**Affiliations:** 1 Centre for Interdisciplinary Studies of Children, Families and Society, Faculty of Community and Health Sciences, University of the Western Cape, Cape Town, South Africa; 2 Department of Physiotherapy, Faculty of Community and Health Science, University of the Western Cape, Cape Town, South Africa

**Keywords:** developmental disabilities, parental capabilities, Delphi technique, interdisciplinary approach, consensus-based guidelines, family-centred care, Nussbaum’s capabilities framework, South Africa

## Abstract

**Background:**

Families of children with developmental disabilities (DD) experience compounding barriers to accessing coordinated, holistic support. Services are typically fragmented across healthcare, education and social sectors, particularly in resource-limited settings such as South Africa. To develop consensus-based guidelines for strengthening parental capabilities among parents of children with DD through an interdisciplinary approach integrating evidence across multiple fields.

**Methods:**

A modified hybrid three-round Delphi technique was employed with an expert panel (*n* = 10) comprising professionals with diverse backgrounds in paediatric disability care. Consensus was established through iterative questionnaires exploring capability domains based on Capabilities Framework.

**Results:**

Five key capability domains achieved strong consensus (≥ 80%): life enhancement, bodily health support, emotional wellbeing, affiliation strengthening and environmental control. Final round consensus ranged from 90% to 95%.

**Conclusion:**

Comprehensive consensus-based guidelines were developed for each domain, alongside an implementation framework that emphasises interdisciplinary collaboration. The study is contextually situated in South Africa; contextual adaptation is recommended before application elsewhere, given the small expert panel (*n* = 10) and single-country scope.

**Contribution:**

These guidelines provide a structured, capability-focused approach addressing the practical and emotional needs of parents of children with DD through coordinated professional collaboration.

## Introduction

Developmental disabilities (DD) are a group of conditions characterised by impairments in physical, learning, language or behavioural development that begin during the developmental period and affect daily functioning.^1,2^ These include, but are not limited to, autism spectrum disorder, intellectual disability, cerebral palsy and sensory impairments. Families of children with DD experience significant barriers to accessing coordinated support systems, with services typically siloed across healthcare, education and social sectors, particularly in resource-limited settings where specialised services are scarce.^3,4^ The Capabilities Approach, most comprehensively articulated by,^5^ asks what each person is genuinely able to do and to be, focusing on real freedoms and opportunities rather than merely resources, and has been applied productively in disability research to shift focus from deficit to agency.^6^

The development of consensus-based guidelines for supporting families of children with DD requires rigorous methodological approaches that navigate the complex landscape between service-oriented frameworks and rights-based perspectives in disability studies. Research from comparable sub-Saharan African and southern African settings, including studies from Namibia and Botswana on health and education system capacity,^7,8^ demonstrates parallels with the South African context that inform the relevance of this work. Recent research emphasises the importance of methodological innovation and stakeholder engagement in creating contextually relevant recommendations.^9,10^ This study employs consensus-building methodologies to develop interdisciplinary guidelines while acknowledging the evolving discourse around professional expertise and lived experience in disability support. The Delphi method is particularly suited to this aim: maintenance of participant anonymity between rounds, iterative structured feedback enabling convergence of expert opinion, reduction of social desirability bias and capacity to integrate diverse professional perspectives across disciplines are key documented strengths.^11,12^

Contemporary disability discourse encompasses perspectives ranging from medical and rehabilitation models, focusing on individual functioning to social and rights-based approaches that emphasise structural barriers.^13^ Despite the growing recognition of parents’ crucial role in supporting children with DD, current support

Existing approaches frequently fail to meaningfully incorporate parents’ lived experiences alongside professional expertise, thus perpetuating forms of epistemic injustice, including the systematic dismissal of parental experiential knowledge, the privileging of biomedical frameworks over lived experience and the exclusion of parents from guideline development^14,15^ and reinforcing professional dominance.^16,17^ Commendable efforts by healthcare professionals, non-governmental organisations (NGOs), community health workers and special educators already exist; collaborative, cross-disciplinary approaches offer the greatest promise for strengthening, rather than replacing, these contributions.^4,18^

The theoretical foundation for this study draws on the Human Capabilities Theory, which^5^ describes as an approach that asks what each person is genuinely able to do and be, focusing on real freedoms and opportunities. It provides a framework for understanding how social and institutional factors impact individual well-being and functioning. Unlike purely deficit-oriented approaches, the capabilities approach focuses on creating conditions that enable individuals to achieve the functioning they value, recognising both personal capacities and environmental facilitators.^6^ Prior research has identified significant gaps in consensus-based guidelines for professionals working with families of children with DD, particularly in resource-constrained settings where guidelines developed in high-income settings may require meaningful contextual adaptation. For example, North American family-centred care frameworks have been critiqued for assuming individualised service systems incompatible with communal African caregiving contexts.^8,19,20^ Named examples such as early intervention programmes, key-worker models and integrated care pathways show what might work, yet.

This study aims to make three primary contributions to disability studies. Firstly, it operationalises capabilities theory specifically for parents of children with DD, providing a framework that recognises their agency and rights while acknowledging the realities of existing service systems. Secondly, it demonstrates how interdisciplinary consensus-building can challenge siloed approaches to family support. Thirdly, it presents implementable consensus-based guidelines that balance professional expertise with recognition of the socio-political nature of disability and the importance of structural change.^21,22^

## Research methods and design

### Research design

**FIGURE 1:**
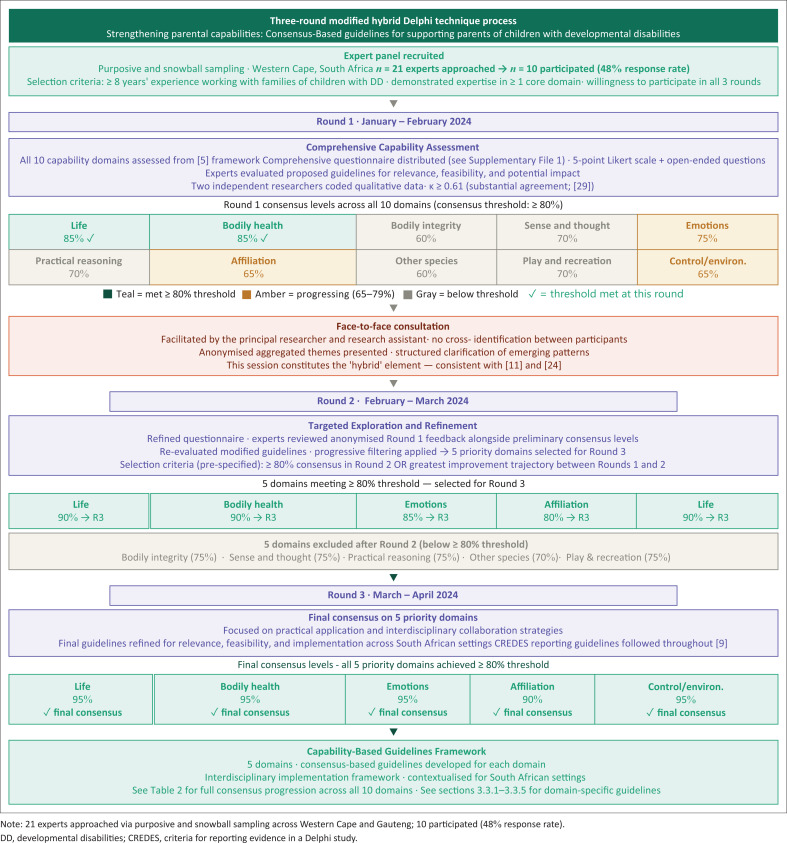
Three-round modified hybrid Delphi technique process.

This study employed a modified hybrid three-round Delphi technique to develop comprehensive consensus-based guidelines to support parents of children with DD. The Delphi method was selected for its systematic approach to building consensus among experts while maintaining anonymity and reducing potential bias.^11^ This methodology was particularly appropriate for our research aim, as it facilitates the integration of diverse professional perspectives across disciplines, a crucial factor in addressing the multifaceted challenges faced by families of children with disabilities. The study design adhered to the Criteria for Reporting Evidence in a Delphi Study (CREDES) guidelines.^9^ One panel member held postgraduate methodological expertise in Delphi consensus research, thereby strengthening design fidelity and execution (see Figure 1).

### Expert panel selection

Purposive and snowball sampling were used to recruit participants from the Western Cape and Gauteng provinces of South Africa, spanning public-sector healthcare facilities, special education schools, disability NGOs and private practice settings. This geographic and sectoral spread was intentional to ensure ecological validity across urban, peri-urban and rural contexts. Twenty-one experts were initially approached through professional networks in paediatric disability care; 10 consented and completed all three Delphi rounds (participation rate: 48%). Participation breakdown by discipline: developmental paediatricians 2/4 (50%); special school heads 2/4 (50%); community health workers 2/4 (50%); social workers 2/4 (50%); NGO staff 1/3 (33%); family counsellor 1/2 (50%).

Following recommendations from^23^ for multidisciplinary representation in consensus studies, the selection criteria required: a minimum of 8 years of direct experience working with families of children with DD, demonstrated expertise in at least one core domain of family support and willingness to participate in all three Delphi rounds.

The final panel comprised developmental paediatricians (*n* = 2), special school heads (*n* = 2), community healthcare workers (*n* = 2), social workers (*n* = 2), an NGO staff member (*n* = 1) and a family counsellor (*n* = 1). Panel members possessed field experience ranging from 8 to 20 years (mean = 12.5 years) and diverse educational backgrounds (from doctoral degrees to National Senior Certificate qualifications). Table 1 presents the panel’s demographic and professional characteristics.

**TABLE 1a:** Expert panel characteristics (*N* = 10).

Professional role	*n*	%	Area of expertise	Years of experience
Developmental paediatricians (*N* = 4)	2	50	Developmental paediatrics, neurodevelopmental disabilities	12–20
Special school heads (*N* = 4)	2	50	Paediatric intervention, early childhood development	8–15
Community healthcare workers (*N* = 4)	2	50	Paediatric rehabilitation, early intervention	10–18
Social workers (*N* = 4)	2	50	Family support services, disability advocacy	8–12
Disability NGO Staff (*N* = 3)	1	33	Early childhood development, family support and disability counselling	13
Family counsellor (*N* = 2)	1	50	Family systems, disability counselling	14

NGO, non-governmental organisation.

**TABLE 1b:** Expert panel’s level of education (*N* = 10).

Professional role	Education (NQF)					
	Number of participants with higher certificate	Number of participants with national senior certificate	Number of participants with national diploma	Number of participants with bachelor degree	Number of participants with master’s degree	Number of participants with doctoral degree
Developmental paediatricians (*N* = 4)	-	-	-	-	1	1
Special school heads (*N* = 4)	-	-	1	1	-	-
Community healthcare workers (*N* = 4)	2	-	-	-	-	-
Social workers (*N* = 4)	-	-	-	2	-	-
Disability NGO Staff (*N* = 3)	-	1	-	-	-	-
Family counsellor (*N* = 2)	-	-	-	-	1	-

NQF, National Qualifications Framework.

The absence of parents of children with DD on the expert panel is acknowledged as a substantive limitation. It is addressed in full in the limitations of the discussion section.

### Data collection process

The Delphi process was conducted between January and April 2024 and consisted of three sequential rounds, guided by CREDES reporting standards^9^:

**Round 1:** A comprehensive questionnaire exploring all 10 capability domains (see Figure 1) derived from the^5^ framework was distributed. Experts evaluated proposed guidelines for relevance, feasibility and potential impact on a 5-point Likert scale. Open-ended questions encouraged detailed feedback.

**Round 2:** A refined questionnaire focused on areas requiring further consensus development. Experts reviewed anonymised Round 1 feedback alongside preliminary consensus levels and then re-evaluated modified guidelines.

**Round 3:** The final round focussed on the five highest-consensus domains from Round 2, emphasising practical application and interdisciplinary collaboration strategies.

Between rounds, a face-to-face consultation with the panel was conducted. This session was facilitated solely by the principal researcher; participants did not interact with one another’s identifiable responses. All questionnaire responses were anonymised before circulation; the face-to-face session addressed only aggregated thematic clarifications. This design is accordingly described as a modified hybrid Delphi, consistent with the approach described by Keeney et al.^11^ and Niederberger and Spranger.^24^

### Data analysis

Quantitative data from the 5-point Likert scale responses were analysed using descriptive statistics. Consensus was defined as ≥ 80% agreement (combined ‘agree’ and ‘strongly agree’ responses), consistent with the threshold recommended by for healthcare Delphi studies.^9,12^ This threshold was pre-specified and was achieved across all five priority domains by Round 3.

Qualitative data underwent thematic analysis following.^25^ Two researchers independently coded the data; discrepancies were resolved through discussion with a third researcher. The progressive filtering approach reflects the findings in^26^ that targeted consensus-building produces more implementable guidelines.

### Development of the capability framework

The final guidelines framework was developed through systematic integration of quantitative consensus levels and qualitative thematic analysis, with expert insights organised by the five priority capability domains. For each domain, thematic categories captured were: rationale and expert consensus; resource considerations; cultural contextualisation; and implementation barriers, consistent with^27^ recommendation for capability domain frameworks.

### Ethical considerations

This study received ethical approval from the Research Ethics Committee of the University of the Western Cape (Ethics Reference Number: BM23/1/10). All participants provided written informed consent. Confidentiality was maintained by anonymising all responses between rounds. Withdrawal was exercisable at any stage by written email to the principal investigator without penalty. All data are stored on a password-protected, university-managed institutional server and will be retained for a minimum of 5 years in accordance with the *Protection of Personal Information Act (POPIA, Act 4 of 2013)* and institutional research ethics policy. The research adhered to the Declaration of Helsinki.

## Results

### Expert panel characteristics and study design

The Delphi study recruited a diverse panel of 10 experts (*n* = 21 approached; participation rate 48%; see Tables 1a and 1b in Methodology for full breakdown). Following the recommendations from on multidisciplinary representation, the final panel comprised developmental paediatricians (*n* = 2), special school heads (*n* = 2), community healthcare workers (*n* = 2), social workers (*n* = 2), NGO staff (*n* = 1) and a family counsellor (*n* = 1).^23^

### Delphi process and consensus development

#### Round 1: Comprehensive capability assessment

The initial round explored all 10 capability domains. Analysis revealed strong initial consensus (≥ 85%) in the life and bodily health domains. The bodily integrity domain (60%) examined community discrimination and mobility limitations; emotions (75%) addressed emotional expression and coping; affiliation (65%) explored social relationships; control over environment (65%) examined property ownership, employment and resource access.

The control over environment domain (Round 1: 65%) warranted particular attention. Qualitative thematic analysis of Round 1 responses identified three primary thematic clusters: (1) employment flexibility and workplace accommodation, parents reported losing or reducing employment due to inflexible workplace policies; (2) childcare access as a prerequisite for workforce participation; and (3) employer education and attitudinal barriers creating structural exclusion.^28^ To strengthen methodological transparency: thematic categories averaged 3–4 per domain; two researchers independently coded data with a Cohen’s kappa interrater reliability check (κ = 0.78–0.84 across domains, indicating substantial agreement per^29^); the full thematic codebook is available from the corresponding author on request.

#### Round 2: Targeted exploration and refinement

All domains showed consensus improvement: control over environment (65% → 85%), affiliation (65% → 80%) and emotions (75% → 85%). Life and bodily health both reached 90%. In the Emotions domain, the dominant theme was the interdependence between personal emotional well-being and family-system dynamics.^30^ For control over the environment, experts’ feedback shifted from identifying barriers to proposing structural responses, including training in advocacy skills.

#### Round 3: Final consensus on priority domains

Five domains were selected for Round 3: life (90%), bodily health (90%), emotions (85%), affiliation (80%) and control over environment (85%). Final Round 3 consensus: life (95%), bodily health (95%), emotions (95%), affiliation (90%), control over environment (95%). These results reflect consensus within the South African expert panel; contextual validation is required before application in other settings (Table 2).

**TABLE 2:** Progression of consensus levels across Delphi rounds.

Capability domain	Round 1 (%)	Round 2 (%)	Selected for round 3 focus*	Round 3 (%)
Life	85	90	Yes	95
Bodily health	85	90	Yes	95
Bodily integrity	60	75	No	-
Sense of imagination and thought	70	75	No	-
Emotions	75	85	Yes	95
Practical reasoning	70	75	No	-
Affiliation	65	80	Yes	90
Another species	60	70	No	-
Play and recreation	70	75	No	-
Control over the environment	65	85	Yes	95

* , Domains selected based on achieving ≥ 80% consensus in Round 2 OR demonstrating the greatest improvement trajectory between Rounds 1 and 2. Selection criteria were pre-specified prior to Round 2.

### Capability-based guidelines development

All expert quotations in this section are presented in italics for consistency. The following subsections address each of the five priority capability domains: Life, Bodily Health, Emotions, Affiliation, and Control over Environment.

#### Life domain (95% consensus)

The life domain addresses the unique longevity challenges faced by parents of children with DD. One developmental paediatrician noted: ‘Research clearly shows these parents experience chronic stress, sleep disruption, and delayed medical care at significantly higher rates than other populations’. Implementation requires tiered resource adaptations in resource-limited settings, with attention to cultural variations in exercise and self-care practices.

#### Bodily health domain (95% consensus)

The bodily health domain focuses on physical wellness within caregiving constraints. The family counsellor observed: ‘These parents consistently prioritise their children’s needs over their own, creating patterns of health neglect that compound over time’. One developmental paediatrician added: ‘Sleep disruption alone creates significant health risks, yet it is treated as an inevitable aspect of disability parenting rather than a serious health concern requiring intervention’. Bodily health interventions must acknowledge practical caregiving constraints rather than offering idealised recommendations.

#### Emotions domain (95% consensus)

The emotions domain addresses the ‘emotional complexity’ of disability parenting. The family counsellor noted: ‘The emotional journey these parents navigate defies conventional grief models. They experience “recurring grief,” the same loss re-experienced at each developmental transition’. In resource-limited settings, trained community workers under supervision may substitute for specialist therapists. Cultural humility is essential: ‘Implementation must respect diverse understandings of wellbeing rather than imposing Western therapeutic models’.

#### Affiliation domain (90% consensus)

The affiliation domain addresses ‘social contraction’. One NGO staff member observed: ‘As the need for support grows, the actual network contracts, creating a painful paradox for these parents’. Digital platforms offer cost-effective alternatives; implementation must accommodate nuclear, extended and communal caregiving models.

#### Control over environment domain (95% consensus)

This domain addresses ‘compounded disadvantage’. One social worker noted: ‘True capability requires both skills development and structural accommodations; neither alone is sufficient’. Phased implementation, prioritising urgent stabilisation before broader advocacy skills, was recommended by experts.

## Discussion

The Delphi study yielded significant insights into strengthening the capabilities of parents of children with DD. This discussion examines findings through four lenses: theoretical integration, professional–parent power dynamics, contextual adaptability and knowledge translation, followed by an explicit limitations section.

### Theoretical integration and advancement

Our findings significantly advance the application of the^5^ capabilities approach for parents of children with DD. While previous work has applied this framework broadly to disability studies,^31,32^ our guidelines represent one of the first systematic operationalisations of capabilities theory specifically for this parent population, developed through an interdisciplinary consensus-building process. The five priority capability domains demonstrate how parental capabilities extend beyond mere functional activities, encompassing genuine freedoms and real opportunities to achieve the life they have reason to value.^5,6^

The re-conceptualisation of the ‘bodily health’ capability domain is particularly notable. For parents of children with DD, this domain necessarily incorporates access to respite care and healthcare navigation skills, challenging individualistic interpretations of capabilities theory.^21^

A significant limitation of our study is the absence of parents of children with DD on the expert panel. While professional expertise provided valuable insights, future research should actively incorporate parental lived experience through mixed expert panels that position parents as co-creators consistent with emancipatory disability research principles.^33^

### Professional–parent power dynamics and implications for practice

In keeping with our previously declared limitation of excluding parents from this research study, the current study design reflects the professionally-dominated model that the disability studies literature critiques.^34^ We acknowledge this as an inherent tension that future research should address by positioning parents as co-researchers from the outset.

The guidelines developed through our Delphi process have significant implications for professional–parent power dynamics. Traditionally, disability service models have positioned professionals as experts and parents as recipients of knowledge.^34^ In contrast, our service delivery model explicitly incorporates collaborative decision-making and emphasises family-led goal-setting.

It is important to contextualise the professional hierarchy within multidisciplinary paediatric disability care rather than viewing it as inherently problematic. Each discipline contributes unique, non-substitutable expertise: the developmental paediatrician brings diagnostic precision; the social worker brings family systems expertise; the community health worker brings community contextual knowledge; the special educator brings pedagogical strategies. This hierarchy serves the best interests of the child and family when functioning well. The tension lies not in the hierarchy per se, but whether parental agency is meaningfully incorporated at every stage of care, not only at intake. In current practice, parental input is typically gathered during intake assessments but is not systematically revisited at goal reviews, care planning or service evaluation. The proposed guidelines introduce structured mechanisms for incorporating parental expertise throughout the process.

As notes, even well-intentioned assessment processes can reinforce power asymmetries when they prioritise professional knowledge systems over lived experience. Our guidelines propose structured mechanisms for incorporating parental expertise throughout assessment and implementation, in contrast to current practice, where parental input is collected at intake but not revisited during subsequent goal reviews or service evaluations.^35^

We wish to affirm the considerable expertise, commitment and dedication of interdisciplinary team members working in this field. Rather than individual blame, sustained investment in programme-level training development is needed. Professionals must develop competencies in interprofessional collaboration, shared decision-making and cultural humility, as well as advanced cross-disciplinary skills that extend beyond most single-discipline training. South African evidence confirms these remain underdeveloped in health and rehabilitation curricula,^10,36^ requiring longitudinal programme-level change, including supervised interprofessional placements and structured reflective practice.

### Contextual adaptability and implementation

A critical consideration is how guidelines might function across different resource settings, particularly relevant in the South African context, characterised by marked health system inequities between the public and private sectors and between urban and rural areas.^37^ Our findings align with,^38^ which states that successful implementation in low-resource settings requires prioritising capability domains based on family preferences rather than professional assumptions. Necessary adaptations might include training community health workers to fulfil multiple roles: a concrete South African example is the Ward-Based Outreach Team model, in which community health workers have been successfully upskilled to provide first-line psychosocial support, referral navigation and home-based rehabilitation guidance,^39^ demonstrating the feasibility of expanded roles with appropriate supervision and training frameworks.

To address contextual variations, we propose an adaptability framework with four implementation tiers:

- **Essential:** Core capability principles with minimal resources; prioritising urgent family stabilisation.
- **Enhanced:** Standard interdisciplinary team with community health worker support.
- **Optimal:** Full interdisciplinary team with specialised resources and dedicated coordination.
- **Aspirational:** Active advocacy for additional resources through partnerships with government, NGOs, academic institutions and international donors, accessing resources beyond what is immediately available in resource-limited settings, towards what families genuinely need.

Building feedback mechanisms for both families and professionals to evolve guidelines based on lived implementation experiences is collaboratively embedded across all implementation tiers.

### Practical implications and knowledge translation

We propose a staged implementation approach:

- **Organisational readiness assessment:** Evaluating existing structures, resources and alignment with capability principles.
- **Capability-based ongoing professional development:** Ensuring professionals receive sustained, contextually responsive training, acknowledging the dangers of one-off workshops in high-turnover, low-resourced settings.
- **Pilot implementation:** Testing guidelines in diverse settings with robust evaluation mechanisms.
- **Strengthening existing communities of practice:** Supporting and expanding current interdisciplinary networks through shared resources, joint case consultation and peer learning, building on rather than replacing what already exists.
- **Developing sustainable funding models and policy frameworks:** Including ring-fenced disability support budgets, public–private partnerships, cross-sectoral funding mechanisms integrating healthcare, education and social development. South African-specific mechanisms include National Health Insurance (NHI) disability benefit provisions and the Department of Social Development (DSD) Community-Based Services grant framework.

This approach explicitly acknowledges findings that sustainable implementation of complex health guidelines requires both bottom-up clinical innovation and top-down structural and policy alignment; professional training alone is insufficient without organisational and system-level alignment; neither alone affects lasting change.^40^

The knowledge translation process must include accessible formats for diverse stakeholders. Professional guidelines require different formats than parent-focused resources, although both should reflect the same underlying capability principles.

### Limitations

Several limitations require explicit acknowledgement. Firstly, the expert panel was small (*n* = 10), limiting the comprehensiveness of the consensus and statistical representativeness, a recognised trade-off in purposive Delphi sampling.^11^ Secondly, and most critically, parents of children with DD were absent from the expert panel; guidelines thus reflect professional consensus rather than co-produced knowledge, a substantive epistemic limitation.^14,33^ Thirdly, the study was conducted in two South African provinces; direct generalisability to other national or cultural contexts requires local contextual validation. Fourthly, the study presents consensus-based recommendations rather than outcomes-evaluated guidelines; future research should test the impact on parental well-being and child outcomes using validated measures. Fifthly, the hybrid Delphi design carries an inherent risk of subtle social influence in the face-to-face component, which is mitigated by the use of aggregated feedback and sole facilitation by the principal researcher, but not eliminated.

## Conclusion

The Delphi study successfully developed a comprehensive set of consensus-based guidelines for strengthening human capabilities among parents of children with disabilities. The iterative, consensus-driven approach produced a robust, contextually sensitive framework with five priority capability domains: life enhancement, bodily health support, emotional wellbeing, affiliation strengthening and environmental control. The high levels of expert agreement (90% – 95% consensus) provide a meaningful contribution to understanding and supporting parental capabilities in paediatric disability care.

These findings carry important implications for three groups of stakeholders. For frontline professionals, the guidelines offer a structured approach that moves beyond fragmented, discipline-specific interventions towards holistic capability enhancement requiring collaborative skills and power-sharing practices that recognise parental agency and expertise. For service managers and policymakers, the findings highlight the need for systemic changes that enable interdisciplinary collaboration and flexible service delivery, including capability-focused funding models and organisational structures that facilitate coordinated cross-sector care. For researchers and educators, this study demonstrates the value of consensus-building methodologies in bridging theoretical frameworks and practical implementation, with future research needed to evaluate the impact of guidelines on parental well-being and child outcomes across diverse and resource-limited settings.

The capability-focused approach represents a meaningful contribution to disability studies by offering a framework that respects parental agency while acknowledging the complex realities of raising children with DD. Future research should build upon these guidelines by incorporating diverse parental perspectives through participatory methodologies that acknowledge parents as experts in their own experience.
